# Development and characterization of lipid nanocapsules loaded with iron oxide nanoparticles for magnetic targeting to the blood–brain barrier

**DOI:** 10.1007/s13346-024-01587-w

**Published:** 2024-05-13

**Authors:** Juan Aparicio-Blanco, Carlotta Pucci, Daniele De Pasquale, Attilio Marino, Doriana Debellis, Gianni Ciofani

**Affiliations:** 1 Department of Pharmaceutics and Food Technology, Faculty of Pharmacy, Complutense University of Madrid, Plaza Ramón y Cajal, 28040 Madrid, Spain; 2 Smart Bio- Interfaces, Istituto Italiano di Tecnologia, Viale Rinaldo Piaggio 34, 56025 Pontedera, Italy; 3 Electron Microscopy Facility, Istituto Italiano di Tecnologia, Via Morego 30, 16163 Genova, Italy; 4 Institute of Industrial Pharmacy, Complutense University of Madrid, Madrid, Spain

**Keywords:** Magnetic targeting, Neurovascular unit, Cerebral endothelial cells, Pericytes, Brain drug delivery, Phase inversion temperature method

## Abstract

Brain drug delivery is severely hindered by the presence of the blood–brain barrier (BBB)*.* Its functionality relies on the interactions of the brain endothelial cells with additional cellular constituents, including pericytes, astrocytes, neurons, or microglia. To boost brain drug delivery, nanomedicines have been designed to exploit distinct delivery strategies, including magnetically driven nanocarriers as a form of external physical targeting to the BBB. Herein, a lipid-based magnetic nanocarrier prepared by a low-energy method is first described. Magnetic nanocapsules with a hydrodynamic diameter of 256.7 ± 8.5 nm (polydispersity index: 0.089 ± 0.034) and a ξ-potential of -30.4 ± 0.3 mV were obtained. Transmission electron microscopy-energy dispersive X-ray spectroscopy analysis revealed efficient encapsulation of iron oxide nanoparticles within the oily core of the nanocapsules. Both thermogravimetric analysis and phenanthroline-based colorimetric assay showed that the iron oxide percentage in the final formulation was 12 wt.%, in agreement with vibrating sample magnetometry analysis, as the specific saturation magnetization of the magnetic nanocapsules was 12% that of the bare iron oxide nanoparticles. Magnetic nanocapsules were non-toxic in the range of 50–300 μg/mL over 72 h against both the human cerebral endothelial hCMEC/D3 and Human Brain Vascular Pericytes cell lines. Interestingly, higher uptake of magnetic nanocapsules in both cell types was evidenced in the presence of an external magnetic field than in the absence of it after 24 h. This increase in nanocapsules uptake was also evidenced in pericytes after only 3 h. Altogether, these results highlight the potential for magnetic targeting to the BBB of our formulation.

**Graphical Abstract:**

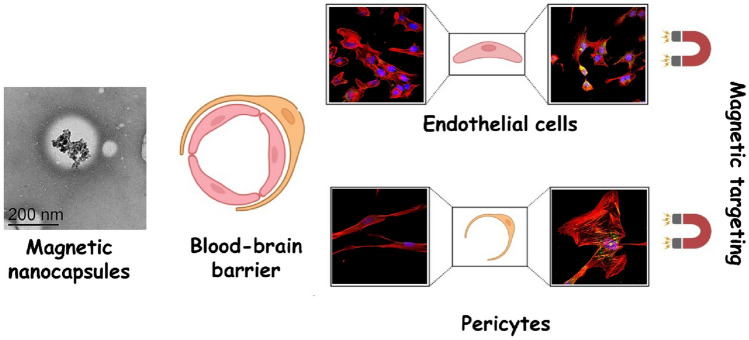

**Supplementary Information:**

The online version contains supplementary material available at 10.1007/s13346-024-01587-w.

## Introduction

The blood–brain barrier (BBB) poses a formidable challenge to drug delivery in the treatment of central nervous system (CNS) diseases. The BBB consists primarily of brain endothelial cells, forming a tight monolayer connected by intricate junctional complexes. Its precise functioning hinges on ongoing interactions with additional cellular constituents, including pericytes, astrocytes, neurons, or microglia, forming altogether the neurovascular unit (NVU) [1]. This cellular arrangement, along with the presence of efflux transporters and the lack of fenestrations, restricts the transport of molecules. While this barrier serves a crucial protective role for maintaining the homeostasis of the CNS microenvironment, it also poses a significant obstacle to brain drug delivery. Hence, effectively delivering drugs across the BBB represents a pivotal challenge in developing treatments for CNS disorders. Some of the most troublesome CNS diseases that could benefit from enhanced brain drug delivery include brain tumours, neurodegenerative diseases such as multiple sclerosis, traumatic brain injury or stroke. The clinical demand for strategies facilitating brain delivery has prompted the exploration of diverse approaches to ensure safe and efficient targeted brain drug delivery. While the most advanced projects have progressed to clinical trials, none has resulted in the launch of new drugs thus far [2].

In this context, nanomedicine emerges as a delivery platform with the potential to elevate the levels of therapeutic agents in the brain by improving the distribution of drugs across the cerebral endothelium [3]. The development of nanocarriers capable of effectively crossing the BBB can be achieved through passive, active, or physical targeting [4]. Passive targeting relies on exploiting the paracellular transport through fenestrations of damaged BBB, while active targeting involves the use of ligands that specifically bind to receptors overexpressed on the brain endothelium to drive BBB crossing. However, designing targeted delivery systems based on either of these inherent biophysical characteristics of the BBB can pose challenges given the heterogeneous idiosyncrasy of the brain vasculature. Alternatively, physical targeting utilizes external stimuli to enhance the delivery of nanoparticles to the brain. Relying solely on physical phenomena, this spatial targeting through external physical stimuli holds promise to address these challenges and streamline the design of nanocarriers [5].

Among physical targeting strategies, magnetic targeting, a technique that leverages magnetic forces to concentrate therapeutic agents at specific sites within the body, has garnered increasing attention for its potential in overcoming the intricately regulated BBB. Magnetic targeting offers a promising strategy to enhance drug delivery across the BBB by exploiting the principles of magnetophoresis. In this approach, magnetically responsive nanocarriers are guided by external magnetic fields, facilitating their accumulation at specific target sites while preventing the cerebral blood flow from dislodging the nanoparticles from their adhesion on the brain endothelium. Such drug localization enhances the concentration gradient favoring cellular internalization, thereby improving the overall efficiency of brain drug delivery. Brain magnetic targeting has already demonstrated to achieve significantly enhanced therapeutic effect in distinct animal models of disease, including glioma [6–10], bacterial meningitis [11] and neurolisteriosis [12].

One of the critical considerations in magnetic targeting to the BBB is the choice of magnetically responsive carriers since magnetic drug delivery systems must rely on strong magnetic moments for enhanced accumulation by external magnetic fields. These carriers should possess biocompatibility and the ability to encapsulate drugs. Magnetic nanoparticles, such as iron oxide nanoparticles, have been extensively investigated for their suitability in this context due to their excellent magnetic properties [13], although their clinical translation for magnetic targeting remains challenging [14]. Some challenges for their clinical translation are related to the safety concerns from previous clinical data using iron oxide nanoparticles for imaging and to the optimization of currently available magnetic devices to achieve deeper and more precise particle control [5, 15, 16]. To address the first challenge, iron oxide nanoparticles are often coated with biocompatible biomaterials, whereas to address the second challenge, intense research is being conducted in developing innovative magnetic devices, including wearable magnetic helmets for brain magnetic targeting obtained by 3D printing [6].

Moreover, the encapsulation of iron oxide nanoparticles within more sophisticated nanocarriers has also been described for brain magnetic targeting purposes [6–9, 11, 17, 18]. Some of the advantages of this encapsulation of iron oxide nanoparticles within nanocarriers include the improved aqueous dispersibility and biocompatibility, together with the reduced tendency of iron oxide nanoparticles to aggregate, along with the possibility to drive a larger number of iron oxide nanoparticles simultaneously and to co-encapsulate one or even various drugs. However, a reduction in saturation magnetization typically occurs upon addition of nonmagnetic components to the iron oxide nanoparticles. Therefore, the saturation magnetization of the final nanocarriers must be tailored for adequate magnetic responsiveness. In this regard, Al-Jamal et al. demonstrated that the magnetic targeting properties of 200 nm-sized magnetic nanocapsules were directly proportional to the loading of iron oxide nanoparticles and to the strength of the magnetic field applied [19].

Among all potential nanocarriers suitable for encapsulation of iron oxide nanoparticles, lipid nanocapsules are selected herein, the interest of which has already been reported for brain drug delivery [20, 21]. Moreover, lipid nanocapsules are particularly suitable for translational purposes since GRAS excipients are used in their formulation, they have been reported to have high drug loading capacity and are prepared by a low energy method, i.e., the phase inversion temperature method [22].

The interaction of magnetic nanocarriers with the components of the BBB, particularly brain endothelial cells, has been a subject of intense research. However, while brain endothelial cells are a central focus in magnetic targeting to the BBB, the role of other cellular components within the NVU should not be overlooked. Pericytes, for example, play a pivotal role in maintaining the integrity and function of the BBB [23]. These mural cells, closely associated with brain endothelial cells, contribute to the regulation of blood flow and the permeability of the BBB. Despite their significance, the impact of magnetic targeting on pericytes and their potential role in drug delivery to the CNS have been relatively understudied. Understanding the interplay between magnetic carriers and the BBB components is critical for developing safe and effective magnetic targeting strategies for CNS drug delivery, including better tailoring of critical parameters like the saturation magnetization, strength of the external magnetic field and exposure time needed to elicit the maximum targeting efficiency for translational purposes.

In this work, the magnetic targeting ability to the BBB of a lipid-based magnetic nanocarrier prepared by a low-energy method is reported. To achieve magnetic responsiveness, iron oxide nanoparticles were encapsulated within the oily core of the lipid nanocapsules with high loading. The iron oxide nanoparticles and the resulting magnetic nanocapsules have been thoroughly characterized in terms of morphology, crystalline structure, hydrodynamic diameter, ξ-potential, iron content and distribution, and magnetic properties. Then, we delve into their biocompatibility in both cerebral endothelial cells and pericytes and their targeting ability upon exposure to an external magnetic field to ultimately gain insight into the saturation magnetization, magnetic field strength and exposure time needed to trigger a significant magnetic targeting for improved brain drug delivery.

## Materials and methods

### Formulation of magnetic nanocapsules

#### Preparation of the chloroform suspension of oleic acid-coated iron oxide nanoparticles (15%wt.)

Commercial oleic acid-coated iron oxide nanoparticles were utilised (Sigma-Aldrich, 07318) and, before use, 5.8 mL of the commercial heptane suspension of oleic acid-coated iron oxide nanoparticles (corresponding to 34.4 mg of iron oxide) were purified by centrifugation (9,000 rpm, 30 min, 20 ºC) upon mixing with acetone (approximately at a 2:1 volume ratio) to precipitate them and redispersed in chloroform twice. The final pellet was redispersed in 154 µL chloroform.

#### Phase inversion temperature method

Lipid nanocapsules loaded with iron oxide nanoparticles (15% (w/w)) were prepared by the low-energy phase inversion temperature (PIT) method with minor modifications [24]. Briefly, 84.6 mg of polyethylene glycol (15)-hydroxystearate (Kolliphor HS15, Sigma-Aldrich), 7.5 mg of soybean phospholipids with 70% phosphatidylcholine (Lipoid S75, Lipoïd), 102.8 mg of medium-chain triglycerides of caprylic and capric acids (Labrafac lipophile WL1349, Gattefossé), 296.2 mg of MilliQ water (Millipore) were mixed with 154 μL of a chloroform suspension of oleic acid-coated iron oxide nanoparticles (15%wt.). The mixture was gradually heated over the PIT of the system up to 90 °C to melt the lipids. Subsequently, the mixture was progressively cooled down until the PIT (74.8 ºC) was reached. Then, a rapid quench with 500 µL cold MilliQ water was applied to form the suspension of magnetic nanocapsules. Magnetic nanocapsules were maintained at 4 °C for 30 min to stabilize the nanocapsules and then purified by centrifugation (16,000 g, 150 min, 4 °C) and redispersed in Milli-Q water (Millipore) thrice. The final pellet was redispersed in 1 mL of MilliQ water. For biological studies, magnetic nanocapsules were labelled with the fluorescent Vybrant DiO cell-labelling dye (Invitrogen, V-22886) by incorporating in the formulation procedure 10 μL of this dye and then proceeding as previously reported.

### Dynamic light scattering (DLS) and electrophoretic light scattering (ELS) measurements

DLS and ELS measurements were conducted with a Zetasizer NanoZS90 (Malvern Instruments Ltd) to determine the hydrodynamic diameter and ζ-potential of the magnetic nanocapsules, respectively. Measurements were conducted at 25 °C on dispersions at a concentration of 400 μg/mL in ultrapure water. For DLS measurements, the hydrodynamic diameter and the polydispersity index were derived from the correlogram through the CONTIN distribution analysis, while the intensity distribution was obtained through the cumulant analysis. For ELS measurements, both ξ-potential and ξ-deviation were derived from the electrophoretic mobility through the Henry equation. All measurements were performed in triplicate.

### Transmission electron microscopy (TEM) studies

In all cases, samples at a concentration of 100 μg/mL were sonicated for 2 min before imaging. A drop of each sample solution (3 µL) was deposited on a Cu grid (150 mesh) coated with an ultrathin amorphous carbon film. To better visualize the lipid nanocapsules, the sample of magnetic nanocapsules was stained with UranyLess EM stain solution (EMS) for 30 s to enhance the contrast of the lipid components.

#### Electron diffraction

Electron diffraction was performed with a JEOL JEM 1011 transmission electron microscope operated at 100 kV in Selected Area Electron Diffraction (SAED) mode to observe the crystalline nature of the iron oxide in the oleic acid-coated iron oxide nanoparticles. The SAED pattern was processed using the PASAD plugin for Gatan Digital Micrograph software by azimuthal integration and background subtraction [25].

#### TEM imaging

TEM imaging was performed with a JEOL JEM 1011 transmission electron microscope operated at 100 kV in TEM bright field (BF) mode to observe the morphology and size of the oleic acid-coated iron oxide nanoparticles and the magnetic nanocapsules.

#### Energy-dispersive X-ray spectroscopy (EDS)

TEM/Scanning transmission electron microscopy (STEM)-EDS analyses were performed with a JEM-1400Plus transmission electron microscope with thermionic source (LaB6) operated at 120 kV. The EDS data were acquired using Dry SD30GV (JEOL) EDS silicon drift type detector (30 mm^2^ effective area) and analytical double-tilt sample holder.

### Colorimetric determination of iron content

The iron content in oleic acid coated iron oxide nanoparticles and magnetic nanocapsules was determined by the 1,10-phenanthroline-based colorimetric assay following a previously reported protocol [26]. Briefly, 40 μL of a diluted suspension of oleic acid-coated iron oxide nanoparticles or of a suspension of magnetic nanoparticles were separately incubated with 20 μL of hydrochloric acid (37% (v/v), Sigma-Aldrich) at room temperature for 4 h under 100 rpm orbital shaking. Subsequently, 100 μL of an aqueous solution of hydroxylamine hydrochloride (10% (w/v), Sigma-Aldrich) were added to reduce all Fe^3+^ to Fe^2+^. Then, 1.8 mL of a sodium acetate buffer 500 mM (pH 4.5) and 200 μL of an aqueous solution of *ortho*-phenanthroline (0.3% (w/v), ACROS Organics) were added to form a coordination complex with Fe^2+^ with a salmon color. Colorimetry was measured at 510 nm with a UV–vis spectrometer (Lambda45 PerkinElmer) to extrapolate iron concentration in each sample. To quantitatively determine the concentration of iron oxide in the nanoparticles, the extrapolated iron concentration obtained was divided by the weight percentage of iron in the corresponding crystalline form of iron oxide. To calculate the relative weight percentage of iron oxide in the final formulations, the total mass of each formulation was inferred from the weight of its dried residue after the evaporation or freeze-drying of predefined volumes of each suspension, respectively.

### Thermogravimetric analysis (TGA)

TGA was conducted with a Q500 analyzer (TA Instruments) to determine the percentage of iron oxide in oleic acid-coated iron oxide nanoparticles and freeze-dried magnetic nanocapsules. Samples were scanned from 30 to 800 ºC at a heating rate of 10 ºC/min under nitrogen atmosphere (flow rate: 50 mL/min).

### Vibrating-sample magnetometry (VSM)

VSM was conducted with a MicroMag 2900/3900 magnetometer (Lake Shore Cryotronics) to determine the magnetic properties of oleic acid-coated iron oxide nanoparticles and freeze-dried magnetic nanocapsules. Magnetization curves were obtained at room temperature applying an external magnetic field ranging from -10 to 10 kOe.

### Cell lines

Biological tests with the resulting magnetic nanocapsules were conducted in both the human cerebral microvascular endothelial hCMEC/D3 (Merck Millipore, SCC006) and Human Brain Vascular Pericytes HBVP (ScienCell, 1200) cell lines. hCMEC/D3 cells were cultured in EndoGRO-MV-VEGF Complete Culture Media Kit (Merck Millipore, SCME003) supplemented with 1% penicillin–streptomycin (P/S, Gibco). HBVP cells were cultured in Pericyte Medium (PM, ScienCell, 1201).

### Biocompatibility of magnetic nanocapsules

Cell metabolic activity of hCMEC/D3 and HBVP cell lines upon treatment with magnetic nanocapsules was assessed by the WST-1 assay, as previously described [27]. The metabolic activity of cells was assessed at 24 and 72 h after the administration of the magnetic nanocapsules. Briefly, 10^4^ cells/cm^2^ were seeded in 48 well-plates. After overnight incubation, cells were treated with different concentrations of magnetic nanocapsules (10, 50, 100, 300 and 500 µg/mL), whereas untreated cells were used as a control. After 24 and 72 h of incubation, treatments were removed, and cells were incubated with 300 µL of WST-1 reagent diluted (1:20) in complete DMEM without phenol red (Gibco) at 37ºC for 40 min (hCMEC/D3) or 2 h (HBVP) due to their different metabolic rates. Thereafter, absorbance was measured at 450 nm using a Perkin Elmer Victor X3 UV–Vis spectrophotometer. The absorbance of the blank (WST-1 1:20 dilution in phenol red-free DMEM) was subtracted from all measurements. The data were expressed as % of cell metabolic activity with respect to untreated controls. All WST-1 assays were performed in triplicate.

### In vitro targeting studies

The uptake of magnetic nanocapsules by hCMEC/D3 and HBVP cell lines was evaluated in vitro in static conditions. Cells (10,000 cells/cm^2^) were seeded on 24-well black µ-plate ibiTreat (Ibidi) and incubated with EndoGRO and Pericyte complete media, respectively. After 24 h, cells were subsequently incubated with 500 µL of 100 µg/mL of magnetic nanocapsules labeled with Vybrant™ DiO Cell-Labeling Solution (Invitrogen) in complete medium. To study the magnetic targeting efficiency of magnetic nanocapsules upon exposure to an external magnetic field source, their uptake was investigated in the presence or absence of a static magnetic field. For that, a cylindrical permanent Nd-Fe-B magnet with axial magnetization (DN01.35NI00.108, 1170–1220 mT remanence, Italfit Magneti) was placed externally under each well upon fixation through a custom-made multi-magnet support as previously described [28].

Following 3 and 24 h treatment incubation, the cells were rinsed twice with PBS and fixed with 4% paraformaldehyde (PFA, Sigma-Aldrich) in PBS (with Ca^2+^ and Mg^2+^) for 20 min at 4ºC. Then, the cells were incubated with Triton X-100 (1:1000 dilution, Sigma-Aldrich) in PBS (with Ca^2+^ and Mg^2+^) for 10 min at room temperature to permeabilize the membrane. For confocal acquisition, the cells were stained with Phalloidin-TRITC (2.5 ng/ml, Invitrogen) and Höechst 3342 (2 µM, Invitrogen) in PBS (with Ca^2+^ and Mg^2+^) for 2 h at 37ºC. After a final wash with PBS, the plates were stored at 4 °C in the dark.

A C2s confocal microscope (Nikon) was used for confocal microscopy acquisitions. Acquisition parameters were maintained constant for the different acquisitions. Images were analyzed with the NIS-Elements software (Nikon) through a semi-automatic approach as previously reported [29]. Briefly, signals of both the F-actin cytoskeleton and the magnetic nanocapsules were selected upon intensity thresholding. The intersection between the two signals indicated the area of the magnetic nanocapsules associated with the intracellular area. This area was normalized to the number of cells according to the number of stained cell nuclei (Höechst signal) in each confocal image. Results were expressed as nanocapsules area (in pixels) relative to cell number.

### Statistical analysis

Data were analyzed using the GraphPad Prism software (version 10.1.0). For the cell viability experiments, statistical analysis was performed using one-way ANOVA followed by a post-hoc Dunnett multiple comparison test. For the quantitative analysis of the nanocapsules internalization, statistical analysis was performed using two-way ANOVA followed by a post-hoc Šidák multiple comparison test. In all cases, statistical significance was fixed as *: *p* < 0.05, **: *p* < 0.01, ***: *p* < 0.001, ****: *p* < 0.0001. Normality and homocedasticity were assumed in all analyses.

## Results and discussion

### Characterization of oleic acid-coated iron oxide nanoparticles

**Fig. 1:**
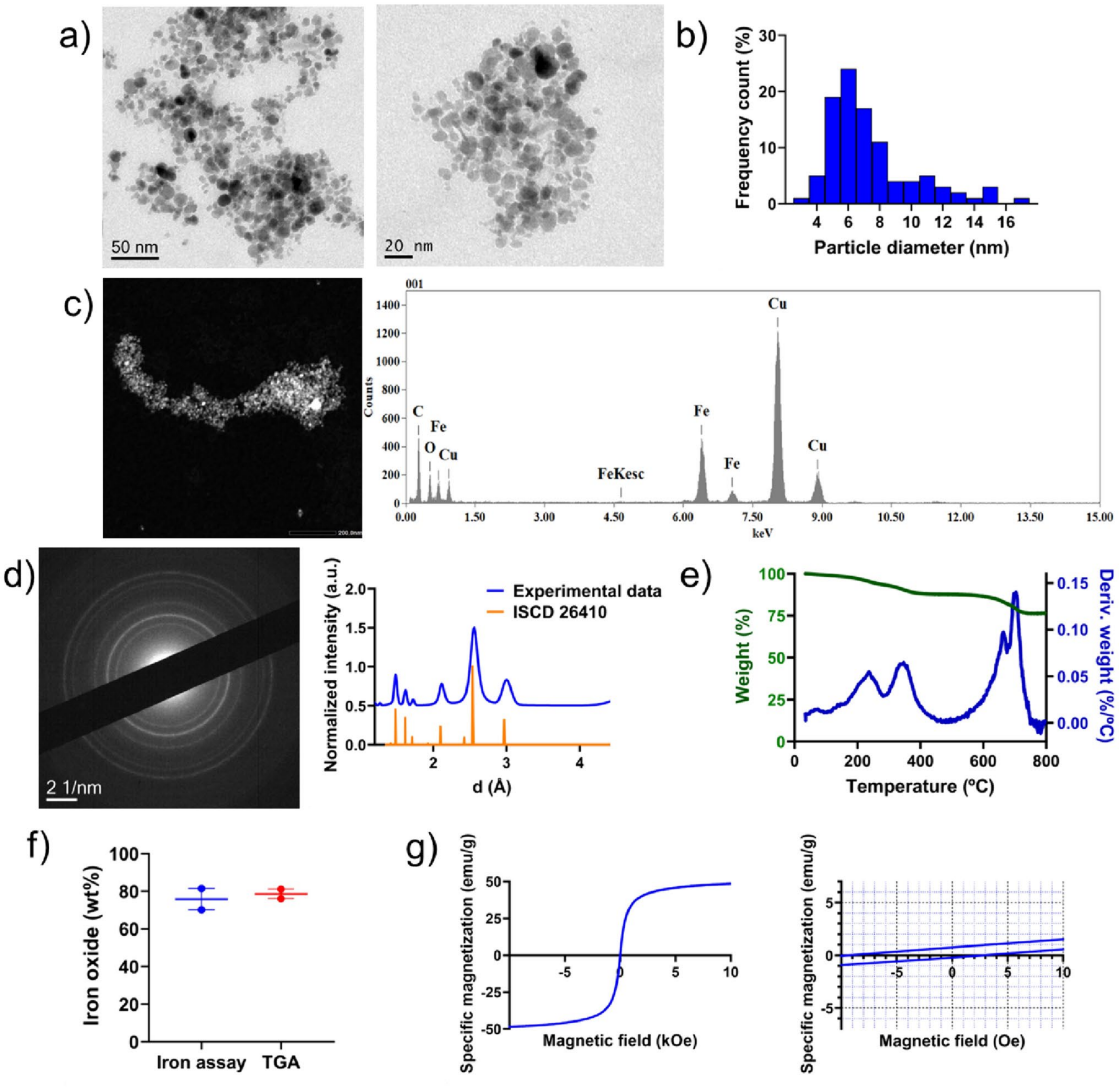
Characterization of oleic acid-coated iron oxide nanoparticles: **a**) Representative transmission electron microscopy (TEM) images in bright field (BF) mode at different magnifications; **b**) Size distribution derived from TEM images; **c**) Chemical mapping using energy dispersive X-ray spectroscopy (EDS) in scanning transmission electron microscopy (STEM); **d**) Selected area electron diffraction (SAED) pattern according to raw data (blue). The obtained intensity profile is compared with a powder X-ray diffraction pattern calculated for the reference ICSD structure magnetite (ICSD 26410, orange); **e**) Thermogram obtained by thermogravimetric analysis (TGA), showing the percentage of weight loss (green) and its derivative (blue) at increasing temperatures; **f**) Comparison of iron oxide weight percentage in oleic acid-coated iron oxide nanoparticles as determined by the 1,10-phenanthroline-based colorimetric assay (blue) and by TGA (red). Results are represented as mean value ± standard error mean (p > 0.05); **g**) Magnetic properties measured by vibrating sample magnetometry (VSM). The figure on the right represents a zoom of the figure on the left at low specific magnetizations and low magnetic fields to evidence the hysteresis loop and determine remanence and coercivity. Remanence can be inferred from the specific magnetization intercept at zero magnetic field, whereas coercivity can be inferred from the magnetic field intercept at zero specific magnetization

The oleic acid-coated iron oxide nanoparticles were first observed by transmission electron microscopy (TEM). As shown in Fig. 1a and b, the iron oxide nanoparticles had a quasi-spherical morphology with a particle size ranging from 5 to 11 nm (average diameter: 7.4 nm, polydispersity index: 0.145). The images also evidenced that the nanoparticles were moderately monodisperse and tended to form aggregates. These results are in agreement with the features of oleic acid-coated iron oxide nanoparticles reported in previous works [30–32].

Then, elemental analysis of the oleic acid-coated iron oxide nanoparticles was conducted through energy-dispersive X-ray spectroscopy (EDS) to further evidence the presence of iron and oxygen in the selected STEM image shown on the left part of Fig. 1c. The EDS data confirmed the presence of C, Cu, O and Fe. The C signal stems from the backbone of the oleic acid, the O signal stems from both the end groups of oleic acid and the oxygen atoms in iron oxide nanoparticles, and the Fe signal stems from the iron atoms in iron oxide nanoparticles. Furthermore, the C and Cu signal are attributable to the TEM grid. In this case, no La or Gd signals were observed as the UranyLess solution was only used to stain lipid samples (see Characterization of magnetic nanocapsules). Altogether, results showed that the elemental analysis was consistent with the expected composition of the oleic acid-coated iron oxide nanoparticles.

Selected area electron diffraction (SAED) was conducted to study the crystalline structure of the oleic acid-coated iron oxide nanoparticles [26, 33]. SAED is a crystallographic technique fundamentally analogous to X-ray diffraction but can serve to examine areas as small as just few hundreds of squared nanometers. The SAED pattern of the oleic acid-coated iron oxide nanoparticles is shown in Fig. 1d. The concentric ring-like diffraction pattern obtained is typical for suspensions of crystalline nanoparticles since the diffraction pattern is made of the 360-degree azimuthal superimposition of spots arising from Bragg reflection from individual nanoparticles. Six characteristic peaks at different crystallographic interplanar distances, namely 1.48760, 1.62175, 1.72528, 2.11733, 2.56123 and 2.99579 Ǟ were observed for the iron oxide nanoparticles. These interplanar distances correspond to the reciprocal radii of each concentric ring in the SAED pattern. This experimental SAED profile matches the position and relative intensity of the peaks of the powder X-ray diffraction pattern calculated for the reference ICSD 26410 structure (magnetite) [34]. In fact, the observed interplanar distances correspond, respectively, to the Miller indices (440), (511), (422), (400), (311) and (220), which define the inverse spinel structure of magnetite. Conversely, this profile does not match with the powder X-ray diffraction pattern calculated for the reference ICSD structure 15,840 (hematite), the other major iron oxide crystal type (Fig. S1).

To calculate the volume of marketed iron oxide nanoparticles to be added during the formulation of lipid nanocapsules, the content of iron oxide in oleic acid-coated iron oxide nanoparticles was first quantified by TGA analysis (Fig. 1e), which demonstrated that the weight percentage of iron oxide with respect to the total nanoparticles weight was 78.60 ± 3.62 according to the remaining final mass at the end of the heating up to 800ºC. These results were supported by the 1,10-phenanthroline-based colorimetric assay. Notably, since SAED diffraction pattern revealed that the nanoparticles consisted of the crystalline structure of magnetite (chemical formula Fe_3_O_4_), to calculate the concentration of iron oxide in the nanoparticles the extrapolated iron concentration from the colorimetric assay was divided by 0.72, which represents the weight percentage of iron in magnetite. The weight percentage of iron oxide in oleic acid-coated nanoparticles was 75.78 ± 8.02. Altogether, averaging colorimetric and TGA results, the oleic acid coating can be assumed to account for ≃ 23% of the total nanoparticle weight (Fig. 1f). Higher [32], analogous [35] and lower [26] weight percentages of oleic acid coating have been reported for other iron oxide nanoparticles. This coating is supposed to favor dispersibility of the iron oxide nanoparticles in organic solvents by preventing particle agglomeration and to enable iron oxide encapsulation within oily phases in nanocapsules.

The magnetic properties of oleic acid-coated magnetite nanoparticles were evaluated by vibrating sample magnetometry (VSM). The left part of Fig. 1g shows the complete magnetization versus magnetic field curve (M-H curve) at room temperature. The saturation magnetization of oleic acid-coated magnetite nanoparticles was 48.63 emu/g. This value is lower than the saturation magnetization of bulk magnetite (85–90 emu/g [36, 37]) but it is in line with the surface effect described for magnetite nanoparticles, wherein a higher proportion of the atoms are near the particle surface where the exchange field is lower, which ultimately accounts for a decrease in magnetization with a decrease in particle size [38–40]. Moreover, the presence of a nonmagnetic coating further decreases the saturation magnetization of iron oxide nanoparticles [41]. Altogether, this saturation magnetization is in the range reported for other oleic acid-coated iron oxide nanoparticles (40–60 emu/g) [9, 30, 42]. The right part of Fig. 1g also shows the M-H curve in the range of ± 10 Oe as an inset to observe the hysteresis loop. With a remanence of 0.48 emu/g and a coercivity of 6.10 Oe, the oleic acid-coated iron oxide nanoparticles behaved as soft ferrimagnets with nearly zero remanence and coercivity. Since the superparamagnetic limit for magnetite state has been estimated to be 25 nm [43], this indicates that, based on their size below this threshold, the thermal energy is enough to randomize the magnetic moments and the magnetite nanoparticles should therefore be in their superparamagnetic state [41]. The superparamagnetic state manifests as a magnetic property that arises in the presence of a magnetic field and disappears upon the removal of the magnetic field. This feature is significant for preventing the interaction of magnetite nanoparticles with iron in biological systems. The small remanent magnetization observed when the magnetic field applied is zero may be accounted for by the presence of a population of blocked nanoparticles due to interparticle interactions in the iron oxide aggregates observed by TEM, as hypothesized in [29]. Overall, the coercivity and remanence values were in agreement with those reported for analogously sized oleic acid-coated magnetite nanoparticles [12, 44].

### Characterization of magnetic nanocapsules

Magnetic nanocapsules were prepared by the low-energy phase inversion temperature method, as previously described elsewhere [24], by further adding a suspension of the oleic acid-coated magnetite nanoparticles in the initial mixture. For biological assays, fluorescently labeled magnetic lipid nanocapsules encapsulating DiO were prepared for particle tracking purposes as indocarbocyanine dyes have been used in lipid-based nanocarriers due to their lipophilic nature and lack of premature release [29, 45, 46]. The characterization features of the resulting magnetic nanocapsules are summarized and compared with those obtained for the oleic acid-coated iron oxide nanoparticles in Table 1.

**Table 1:** Comparison of physicochemical characterization of oleic acid-coated iron oxide nanoparticles and magnetic nanocapsules

	Oleic acid-coated iron oxide nanoparticles	Magnetic nanocapsules
Size (nm)	7.4 (TEM)	256.7 (DLS)
PdI	0.145 (TEM)	0.089 (DLS)
Zeta potential (mV)	-	-30.4 ± 0.3
Zeta deviation (mV)	-	4.99 ± 0.30
Iron oxide weight percentage (%)	75.78 (Colorimetric assay) 78.60 (TGA)	11.18 (Colorimetric assay) 12.77 (TGA)
Saturation magnetization (${M}_{s}, emu/g$)	48.63	5.84
Coercivity (${H}_{c}$, Oe)	6.10	6.60
Remanence (${M}_{r}, emu/g$)	0.48	0.12

**Fig. 2:**
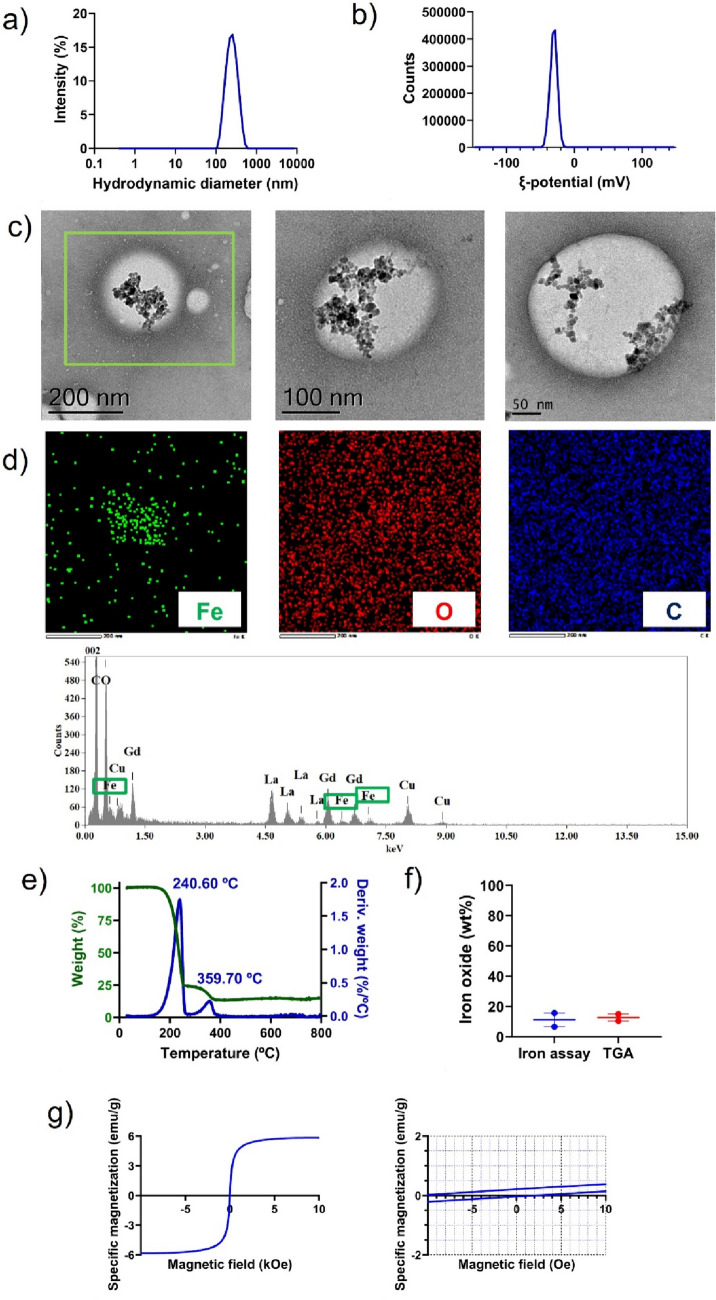
Characterization of magnetic nanocapsules: **a**) Representative intensity distribution profile (%) as a function of the hydrodynamic diameter (nm) measured by dynamic light scattering (DLS); **b**) Representative ζ-Potential (mV) distribution measured by electrophoretic light scattering (ELS); **c**) Representative transmission electron microscopy (TEM) images in bright field (BF) mode at different magnifications; **d**) Chemical mapping of the squared area in the left image of c) using energy dispersive X-ray spectroscopy (EDS) in bright field transmission electron microscopy (TEM): iron (green), oxygen (red), carbon (blue); **e**) Thermogram obtained by thermogravimetric analysis (TGA), showing the percentage of weight loss (green) and its derivative (blue) at increasing temperatures; **f**) Comparison of iron oxide weight percentage in oleic acid-coated iron oxide nanoparticles as determined by the 1,10-phenanthroline-based colorimetric assay (blue) and by TGA (red). Results are represented as mean value ± standard error mean (*p* > 0.05); **g**) Magnetic properties measured by vibrating sample magnetometry (VSM). The figure on the right represents a zoom of the figure on the left at low specific magnetizations and low magnetic fields to evidence the hysteresis loop and determine remanence and coercivity. Remanence can be inferred from the specific magnetization intercept at zero magnetic field, whereas coercivity can be inferred from the magnetic field intercept at zero specific magnetization

To study the hydrodynamic size distribution of the resulting nanocarriers, dynamic light scattering (DLS) measurements were conducted in water. According to the predictive univariate mathematical model defined in [24] for blank lipid nanocapsules prepared by this method, a hydrodynamic diameter of around 50 nm was to be expected for the theoretical initial 1.2 oil/surfactant (i.e., Labrafac WL1349/Kolliphor HS15) weight ratio used for the preparation of the formulation of magnetic lipid nanocapsules, as widely reported experimentally [21, 47–49]. However, as shown in Fig. 2a, after purification by centrifugation, magnetic nanocapsules showed an average hydrodynamic diameter of 256.7 ± 8.5 nm. Hence, in this case, and conversely to what had been previously reported [50, 51], the addition of magnetite nanoparticles greatly increased by approximately fivefold the particle size of their blank lipid nanocapsules counterparts (Fig. S2a). A plausible explanation could be the one given by Cui et al., who directly correlated the growing size of PLGA-based nanoparticles with increasing content of magnetic nanoparticles [9]. Regarding the polydispersity index of magnetic nanocapsules though, a highly monodisperse size distribution was obtained with a polydispersity index of 0.089 ± 0.0034, in agreement with results reported for non-magnetic lipid nanocapsules [48, 52–54]. Previous studies have reported both magnetic lipid-based [55, 56] and polymer-based [8, 17, 57] nanoparticles of 200–250 nm for magnetic targeting purposes to the BBB. Hence, the size distribution of magnetic nanocapsules was deemed to be adequate to benefit from magnetic targeting to the BBB.

The ζ-potential of the dispersion of magnetic nanocapsules in water was measured by electrophoretic light scattering (ELS). As shown in Fig. 2b, magnetic nanocapsules had an average ζ-potential of—30.4 ± 0.3 mV and a ζ-deviation of 4.99 ± 0.30 mV. Slightly negative ζ-potentials had been reported previously for lipid nanocapsules, although the lower ζ-potentials in absolute value may be related to the ionic strength of the dispersion medium, which tends to decrease the ζ-potential [51]. However, the ζ-potential value reported herein correlates with the trend observed in [58], where it was observed that the higher the particle size of lipid nanocapsules, the higher the ζ-potential in absolute value: in particular, 200 nm-sized lipid nanocapsules had a ζ-potential of ≃—20 mV. The observed negative ζ-potential is expected to prevent non-specific adsorption of the lipid nanocapsules to the negatively charged cell membranes [59]. This can ultimately prevent nonspecific systemic uptake from occurring and increase the availability of nanocapsules for magnetic targeting to the BBB.

The ζ-deviation, although not frequently reported in literature, is a measure of dispersion of the ζ-potential distribution. The ζ-deviation value below 5 mV observed for the magnetic nanocapsules is consistent with a highly monodisperse ζ-potential distribution. Overall, the ζ-potential provides information on the electrostatic repulsive forces between the dispersed magnetic nanocapsules. The addition of magnetite nanoparticles did not modify the ζ-potential of their blank lipid nanocapsules counterparts (Fig. S2b), which aligns well with encapsulation of the oleic acid-coated iron oxide nanoparticles within the lipid core of the nanocapsules. A ζ-potential above |30| mV as in this case is often correlated with high colloid stability in the literature as per the electrostatic component of the DLVO theory [60]. Altogether, in terms of surface properties, the magnetic nanocapsules were foreseen suitable for magnetic targeting to the BBB.

Hydrodynamic diameter, polydispersity index and zeta potential of fluorescently labeled magnetic nanocapsules were analogous to the unlabeled magnetic nanocapsules (data not shown).

TEM imaging was performed to observe the morphology and structure of the magnetic nanocapsules. As shown in Fig. 2c, TEM images confirmed the spherical morphology of the lipid nanocapsules, which associated with the brighter regions. The particle size of the magnetic nanocapsules as observed by TEM ranged from 210 to 250 nm, slightly below the hydrodynamic diameter obtained by DLS. This trend was to be expected given that hydrodynamic diameter stems from solvated samples, whereas TEM imaging occurs on dry samples under vacuum conditions, which ultimately often leads to size obtained by DLS being bigger than that observed in TEM images [60]. Within the lipid core of the nanocapsules, electron-dense spots were observed forming grape-like aggregates. The particle size of these electron-dense spots ranged from 8 to 15 nm, which matches with the particle size observed for the oleic acid-coated magnetite nanoparticles under TEM imaging (Fig. 1a, b). Moreover, a significant increase in size was observed for nanocapsules that contained the dark spots in comparison with those devoid of them (as showed on the left image of Fig. 2c). However, artifacts can occur during TEM imaging upon staining with uranyl acetate. Hence, to prevent misinterpretation, elemental analysis was further conducted to verify the efficient encapsulation of the iron oxide nanoparticles within the core of the lipid nanocapsules. Then, elemental analysis through energy-dispersive X-ray spectroscopy (EDS) was conducted to evidence the location of the elements on the lipid nanocapsules in the selected area highlighted in green in Fig. 2c. As shown in Fig. 2d, the EDS analysis revealed the presence of C, O, Fe, Cu, La and Gd. The C signal stems from both the backbone of the polyethylene glycol (15)-hydroxystearate and the medium-chain triglycerides of caprylic and capric acids, the O signal stems from the ethylene oxide monomers of the polymer, from the glycerol moieties of triglycerides and from the oxygen atoms in magnetite, and the Fe signal stems from the iron atoms in magnetite. Moreover, the C and Cu signals are attributable to the TEM grid, whereas La and Gd signals are introduced by the staining UranyLess solution. The Fe peak on the dark particles in the TEM image confirmed that these electron-dense particles were iron-containing particles. Interestingly, the analysis revealed a homogeneous distribution of the C, O and Fe elements on a single nanocapsule. Altogether, the co-localization of Fe signal with the electron-dense particles in the core of the nanocapsules in the corresponding TEM image and with O and C signals in the elemental analysis confirmed the efficient encapsulation of iron oxide nanoparticles within the oily core of the lipid nanocapsules. Overall, this analysis demonstrates more strongly the encapsulation of iron oxide nanoparticles within lipid nanocapsules than the sole two previous studies that attempted to perform likewise [50, 51]. On the one hand, Moura et al. claimed to have iron oxide nanoparticles co-localized within the oily core of the lipid nanocapsules based solely on single and scarce electron-dense spots in TEM images [51]. Instead of using EDS analysis to demonstrate colocalization of Fe signal within the oily core of lipid nanocapsules, this analysis was only used to evidence a functionalization process through the presence of the S signal. On the other hand, Bohley et al. also relied on TEM images where the lipid nanocapsules themselves were entirely electron-dense instead of showing the grape-like morphology usually reported for iron oxide nanoparticles under TEM imaging [50]. Overall, this lack of efficient encapsulation may also account for the fact that neither of the authors did report a significant increase in particle size upon the addition of iron oxide nanoparticles as observed in this study. The encapsulation of oleic acid-coated magnetite nanoparticles within the oily core of lipid nanocapsules is likely a result of, on the one hand, hydrophobic interactions between the oleic acid coating stabilizing the magnetite nanoparticles and the medium chain triglycerides forming the core of the nanocapsules and, on the other hand, of the purification steps by centrifugation that enriches the samples in nanocapsules loaded with magnetite nanoparticles.

As reported for the oleic acid-coated iron oxide nanoparticles, the iron oxide weight percentage in the magnetic nanocapsules was quantified using both TGA and the 1,10-phenanthroline-based colorimetric assay. The weight percentage of iron oxide in magnetic nanocapsules determined by TGA analysis (Fig. 2e) was 12.77 ± 3.21 according to the remaining final mass at the end of the heating up to 800ºC. These results were supported by the colorimetric assay, which demonstrated that the weight percentage of iron oxide with respect to the total nanocapsules weight was 11.18 ± 6.45. Altogether, averaging colorimetric and TGA results, the iron oxide can be assumed to account for ≃ 12% of the total nanocapsules weight (Fig. 2f), which represents a 79.83% encapsulation yield with regards to the theoretical 15% initial iron oxide weight percentage. The increase of the organic component in magnetic nanocapsules in comparison with oleic acid-coated iron oxide nanoparticles from ≃ 23% to ≃ 88% is consistent with the increase in weight percentage due to the polymer shell and oily core included in the formulation of lipid nanocapsules. Indeed, in Fig. 2e, the weight loss curve (in green) and the corresponding derivative weight loss curve (in blue) show that the total weight of the nanocapsules decreases in two steps as the temperature increases due to the thermal decomposition of the different organic excipients (i.e., lipid and polymers), which have distinct decomposition temperatures. The first peak observed in the derivative weight loss curve in Fig. 2e at ≃ 240ºC can be ascribed to medium-chain triglycerides of caprylic and capric acids (Labrafac lipophile WL1349) according to the information provided by the supplier in its safety data sheet. The second peak in the derivative weight loss curve at ≃ 360ºC matches the thermal decomposition temperature reported for polyethylene glycol (15)-hydroxystearate (Kolliphor HS15) by the supplier. Above 360ºC and up to 800ºC there is no further weight loss since the polymeric and lipid excipients have already been thermally decomposed and remaining iron oxide does not degrade at these temperatures. No significant weight loss was observed at temperatures below 100ºC that could be due to water evaporation since samples had been freeze-dried prior to TGA analysis. Notably, the first thermal decomposition event was associated with a weight loss of about 75.8% of the total nanocapsules weight, whereas the second thermal decomposition event was associated with a weight loss of about 12.6%. This may explain why magnetic nanocapsules showed a bigger particle size than expected according to previous studies. In fact, TGA analysis seems to outline that after the purification steps by centrifugation only those nanocapsules loaded with iron oxide nanoparticles and with a bigger size sedimented. This accounts for the fact that despite a theoretical initial 1.2 Labrafac WL1349/Kolliphor HS15 weight ratio was used for the preparation of lipid nanocapsules, the final formulation was formed by a 6.0 mass ratio between both excipients. Applying the linear univariate mathematical model to predict nanocapsules sizes as a function of the Labrafac WL1349/Kolliphor HS15 weight ratio described elsewhere [24], volume diameter of nanocapsules is expected to be approximately 180 nm, which more closely matches the sizes experimentally reported herein. Altogether, the iron oxide weight percentage in the final magnetic nanocapsules is in line with or slightly above than that reported in other studies for nanocarriers prepared for magnetic targeting purposes [35, 55].

To authenticate the feasibility and sensitivity of the developed magnetic nanocapsules as stimuli-responsive nanocarriers, it is important to retain the magnetic properties of the oleic acid iron oxide nanoparticles after encapsulation. Accordingly, the magnetic properties of magnetic nanocapsules were also evaluated at room temperature by VSM. The left part of Fig. 2g shows the complete M-H curve. The saturation magnetization of magnetic nanocapsules was 5.84 emu/g. This value is lower than the saturation magnetization of oleic acid-coated iron oxide nanoparticles. Specifically, the specific saturation magnetization of the magnetic nanocapsules was 12.0% of that of the oleic acid-coated iron oxide nanoparticles, in full agreement with the iron oxide weight percentage in the final formulation as shown by both the colorimetric iron assay and TGA analysis. This reduction in magnetization is therefore a consequence of the weight percentage of nonmagnetic excipients included in the formulation, as also observed in [61]. For a nanocarrier to be suitable for magnetic targeting, high saturation magnetization is needed. Overall, the saturation magnetization of the magnetic nanocapsules is above the saturation magnetization values reported for other nanocarriers prepared for magnetic targeting purposes [6, 55, 56, 62, 63]. As a result, this saturation magnetization value was considered sufficient to provide magnetic targeting responsiveness. Furthermore, lipid nanocapsules become near fully saturated at relatively low magnetic fields (5,000 Oe, equivalent to 0.5 T). The right part of Fig. 2g also shows the M-H curve in the range of ± 10 Oe as an inset to observe the hysteresis loop. Magnetic nanocapsules had a coercivity of 6.60 Oe and a remanence of 0.12 emu/g. In comparison with the values observed for oleic acid-coated iron oxide nanoparticles, the low coercivity value was maintained whereas specific saturation remanence was decreased by fourfold for magnetic nanocapsules, which may be due to the reduced occurrence of iron oxide aggregates upon encapsulation within lipid nanocapsules, as also observed in [29]. Altogether, both near zero coercivity and remanence values contribute to the superparamagnetic-like state of the magnetic nanocapsules. Indeed, Azarmi et al., with higher remanence values for their iron oxide nanoparticles than those reported herein, claimed their particles to be superparamagnetic [12]. Hence, even if the drop in saturation magnetization upon encapsulation of iron oxide nanocapsules in lipid nanocapsules might have been regarded as a caveat to respond to an external magnetic field for magnetic targeting, this encapsulation may likewise help prevent the iron oxide nanoparticles from aggregating contributing thereby to a more superparamagnetic-like behavior.

### Interaction of magnetic nanocapsules with the human cerebral microvascular endothelial hCMEC/D3 cell line

**Fig. 3:**
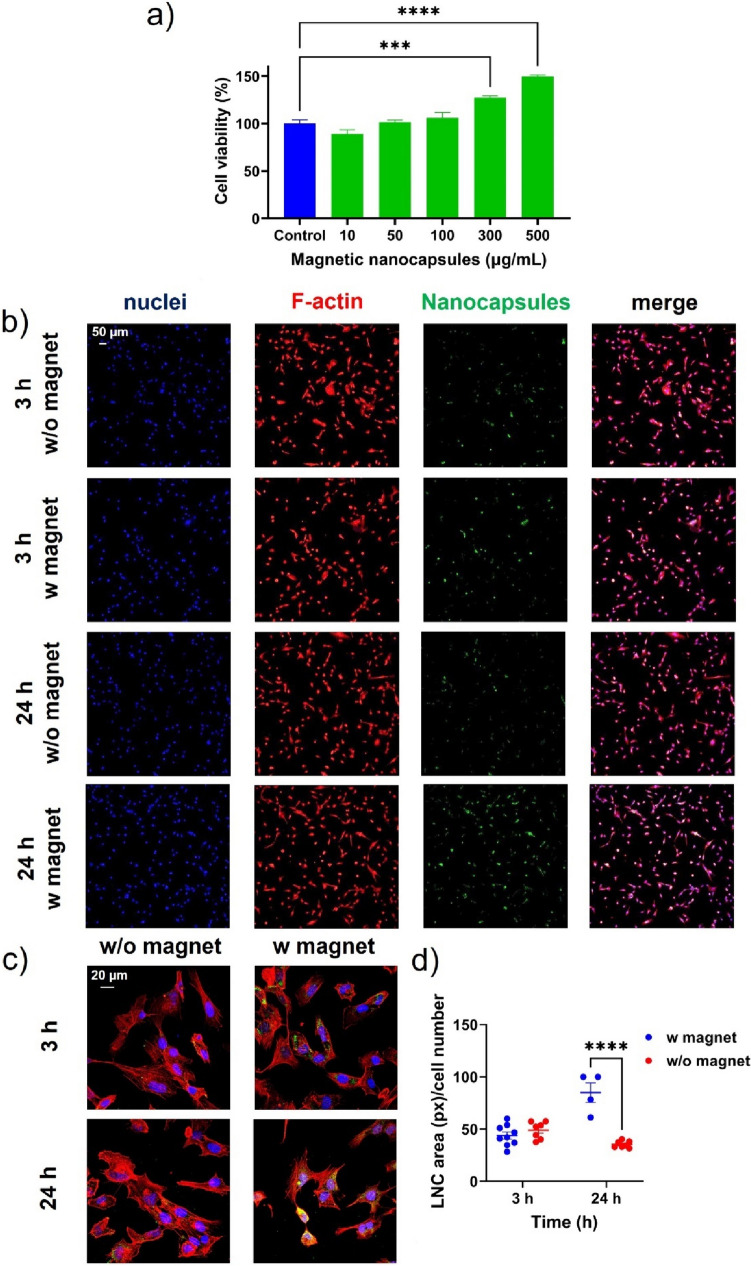
Evaluation of magnetic nanocapsules on the human cerebral microvascular endothelial cell line hCMEC/D3: **a**) Cell viability after 24 h treatment with magnetic nanocapsules (10-500 µg/mL). All results were normalized to untreated control. Statistical analysis was performed using one-way ANOVA and Dunnett multiple comparison test. ***: p < 0.001; ****: p < 0.0001; **b**, **c**) Representative confocal imaging showing the uptake of magnetic nanocapsules (green, 100 µg/mL) by hCMEC/D3 cells after 3 and 24 h treatment in static conditions in the presence (w) and absence (w/o) of a magnetic field at different magnifications; **d**) Quantitative analysis of the magnetic nanocapsules internalization expressed as nanocapsules area (in pixels) relative to cell number. Statistical analysis was performed using two-way ANOVA and Šidák multiple comparison test. ****: p < 0.0001

First, the biocompatibility of the magnetic nanocapsules was tested on the human cerebral endothelial cell line hCMEC/D3 at different concentrations following 24 h (Fig. 3a) and 72 h (Fig. S3a) of treatment. The WST-1 assay was chosen to infer the cytotoxicity profile from changes in cellular metabolic activity. Notably, magnetic nanocapsules did not reduce the metabolic activity of hCMEC/D3 cells at any of the concentrations tested ranging from 10 to 500 µg/mL after 24 h (Fig. 3a). These data may seem controversial in comparison with a recent study on the effect of blank lipid nanocapsules on metabolic activity of this cell line [51]. In that previous study, a significant reduction in cell viability for the highest concentrations of blank lipid nanocapsules was evidenced after 24 h and even after only 4 h treatment. However, the concentration range tested there, when expressed in the same units as those utilized herein, correspond to ≃ 450 µg/mL-≃ 1750 µg/mL. Hence, the highest concentration evaluated in this study is in the same order than the lowest tested in the previous study, which may well account for the higher cytotoxicity observed there. The viability data obtained for the magnetic nanocapsules also aligns well with the results observed for smaller sized blank lipid nanocapsules following 24 h incubation [21]. Nonetheless, straightforward comparisons between lipid nanocapsules concentration cannot be fully drawn given that different excipient weight ratios (with distinct intrinsic toxicities) are used for their formulation in each case. Overall, particle size has been reported to play a role in toxicity of lipid nanocapsules on other cell lines, with the lowest toxicity observed for the biggest particle sizes [58]. This may well be due to the intrinsic toxicity of the surfactant, whose weight ratio decreases as the particle size increases. This reinforces the suitability of 250 nm-sized magnetic nanocapsules for biocompatibility purposes. Importantly, only the concentration of 500 µg/mL reduced in a statistically significant manner the metabolic activity of hCMEC/D3 cells after 72 h (Fig. S3a), which demonstrates the low toxicity of the magnetic nanocapsules in a longer term for this cerebral endothelial cell line. These results seem to outline both a time-dependent and concentration-dependent drop in viability for hCMEC/D3 cells, in agreement with Moura et al. [51]. The partial mismatch between the cell viability observed after 24 and 72 h for the highest concentration tested may arise from lipid samples exhibiting antioxidant effects, which diminish over time. Throughout cell culture, the antioxidant activity exerted by the lipid component of the nanocapsules diminishes over time, concurrent with the gradual release of the iron oxide component, which has the potential to elevate cellular oxidative stress therefore affecting cell viability in the long term*.*

Then, the uptake of the magnetic nanocapsules (at a concentration of 100 µg/mL) by the human cerebral endothelial cell line hCMEC/D3 as the archetype cell type of the BBB was tested at two different time points (i.e., 3 and 24 h, Fig. 3b-d) using confocal microscopy. The cellular uptake was tested in the presence and in absence of an external magnetic field to investigate the magnetic targeting responsiveness of magnetic nanocapsules in static conditions. The magnetic targeting ability was determined by comparing qualitatively (Fig. 3b-c) and quantitatively (Fig. 3d) the fluorescent signal associated with the magnetic nanocapsules internalization extent in the presence and in absence of the external magnetic field. For quantitative analysis the magnetic nanocapsules internalization was expressed as nanocapsules area (in pixels) relative to cell number. The uptake of magnetic nanocapsules was time-dependent only in the presence of the external magnetic field, with a higher internalization extent after 24 h exposure. Notably, statistically significant 2.4-fold higher uptake of magnetic nanocapsules in hCMEC/D3 cells was evidenced in the presence of an external magnetic field than in its absence after 24 h (Figs. 3b-d). However, this increase in uptake was not observed after only 3 h of exposure to the external magnetic field. This time-dependent increase in magnetically targeted cell uptake has been reported previously for this cell line [64].

Previous studies had also evidenced that 4 h exposure to a static external magnetic field had not been enough to significantly increase the uptake of salinomycin-loaded iron oxide nanoparticles by the murine brain endothelial cell line bEnd.3 either [65].The same authors achieved a statistically significant increase in the cell internalization extent of doxorubicin-loaded iron oxide nanoparticles by the bEnd.3 cells after 4 h exposure to a magnetic field but only at the highest concentration tested, which might be well related to the higher saturation magnetization of the nanoparticles at higher concentrations [66]. However, neither the magnetic field strength, nor the saturation magnetization of the nanoparticles, were reported for comparison purposes with the results shown herein for the magnetic nanocapsules in the human brain endothelial cell line. Analogously, Sun et al. only observed significant increase in bEnd.3 internalization upon 5 h exposure to magnetic targeting (1300 mT field strength) for the highest concentrations of iron oxide nanoparticles tested and only when the iron oxide nanoparticles were modified with a positively-charged coating to further enhance cell uptake [33]. Nonetheless, the saturation magnetization of these iron oxide nanoparticles was not reported. Cui et al. observed that magnetic targeting did not significantly boost the cellular internalization of magnetic PLGA nanoparticles by the bEnd.3 cell line after 2 h exposure to a magnetic field [9]. These latter results are accounted for by the fact that the strength of the applied magnetic field (i.e., 100 mT) was 10 times lower than the one used in the current study (1100–1200 mT) and the saturation magnetization of the magnetic PLGA nanoparticles (i.e., 13 emu/g Fe) was approximately 5 times lower than the one used in the current study (5.84 emu/g magnetic nanocapsules corresponds to 63.52 emu/g Fe by considering both the iron oxide content derived from TGA analysis and the 0.72 weight percentage of iron in magnetite).

Altogether, when evaluating the in vitro magnetic targeting abilities of iron oxide nanoparticles-loaded nanocarriers, the exposure time needed is highly dependent on the magnetic field strength and on the saturation magnetization of the nanocarrier, even if these data are often not reported. Altogether, results shown in Fig. 3 highlight the potential for in vitro magnetic targeting to the human cerebral endothelial hCMEC/D3 cell line of the magnetic nanocapsules formulation with a specific saturation magnetization of 5.84 emu/g.

### Interaction of magnetic nanocapsules with the human brain vascular pericytes HBVP cell line

While magnetic targeting functions by locally concentrating magnetically responsive carriers at the target site, enhancing the concentration gradient to facilitate cell internalization, the effectiveness of this approach ultimately hinges on the internalization rate of each cell type. In the context of the BBB, composed not only of brain endothelial cells but also various cell types forming altogether, the NVU, pericytes emerge as pivotal contributors. These mural cells encircle in entire physical juxtaposition nearly 100% of the abluminal surface of the brain endothelium, playing a crucial role in the NVU's barrier function [23].

Despite their central role, pericytes have been largely overlooked in the realm of brain drug delivery. Recognizing their significance, this study explores the interaction of magnetic nanocapsules with pericytes, aiming to shed light on their involvement in the magnetic targeting to the BBB.

**Fig. 4:**
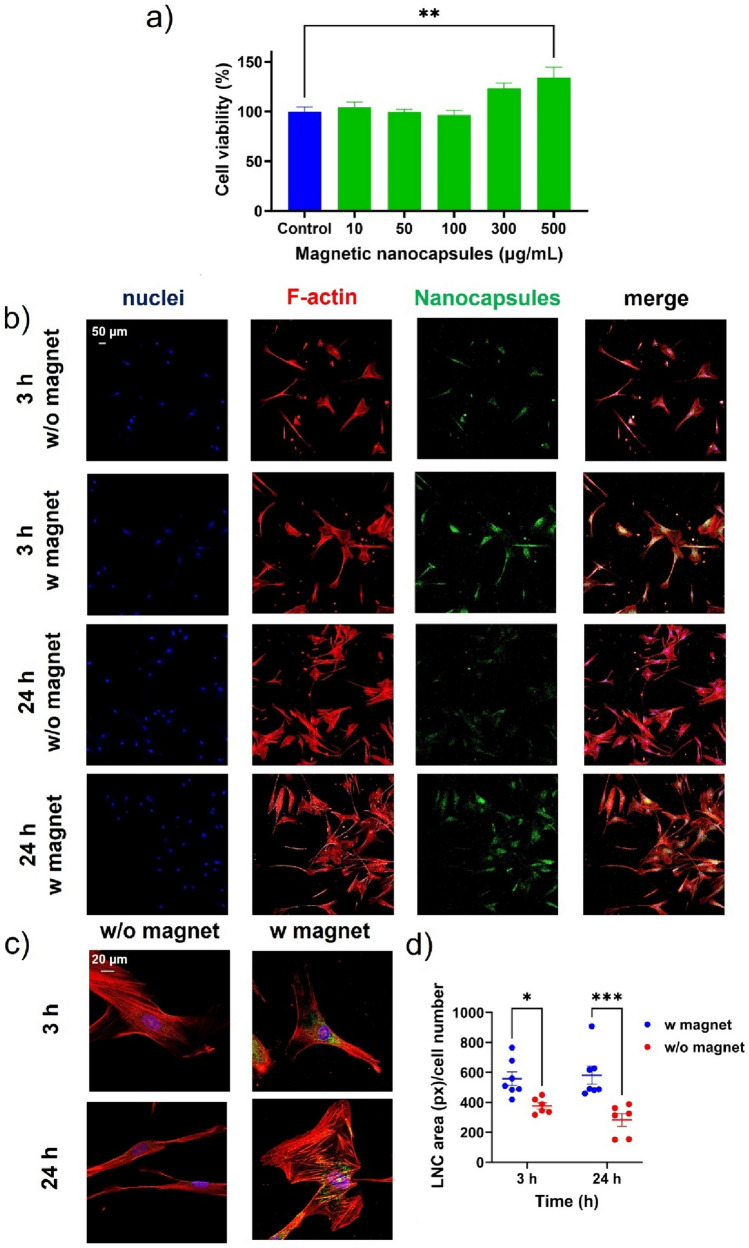
Evaluation of magnetic nanocapsules on the human brain vascular pericytes HBVP cell line: **a**) Cell viability after 24 h treatment with magnetic nanocapsules (10–500 µg/mL). All results were normalized to untreated control. Statistical analysis was performed using one-way ANOVA and Dunnett multiple comparison test. **: p < 0.01; **b**, **c**) Representative confocal imaging showing the uptake of magnetic nanocapsules (green, 100 µg/mL) by HBVP cells after 3 and 24 h treatment in static conditions in the presence (w) and absence (w/o) of a magnetic field at different magnifications; **d**) Quantitative analysis of the magnetic nanocapsules internalization expressed as nanocapsules area (in pixels) relative to cell number. Statistical analysis was performed using two-way ANOVA and Šidák multiple comparison test. *: *p* < 0.05; ***: *p* < 0.001

Initially, the biocompatibility of magnetic nanocapsules was assessed on the HBVP human brain vascular pericytes cell line at various concentrations following 24 h (Fig. 4a) and 72 h (Fig. S3b) of treatment. Notably, the metabolic activity of HBVP cells was not reduced by magnetic nanocapsules across concentrations ranging from 10 to 500 µg/mL after 24 h (Fig. 4a). This favorable viability profile persisted even after 72 h (Fig. S3b), underscoring the low toxicity of magnetic nanocapsules for this specific pericyte cell line. It is noteworthy that, to the best of the authors' knowledge, no previous studies have investigated the effect of lipid nanocapsules on this cell line. For consistency with the in vitro uptake experiments conducted with hCMEC/D3 cells, subsequent experiments with the HBVP cell line employed the same concentration of 100 µg/mL of magnetic nanocapsules.

Analogously, the uptake of the magnetic nanocapsules by the HBVP cell line was evaluated at the same two different time points (i.e., 3 and 24 h, Fig. 4b-d) in the presence and in absence of an external magnetic field to investigate their magnetic targeting responsiveness in static conditions using confocal microscopy. The magnetic targeting ability was determined qualitatively (Fig. 4b-c) and quantitatively in terms of nanocapsules area (in pixels) relative to cell number (Fig. 4d). In this case, neither in the presence nor in absence of the external magnetic field was the uptake of magnetic nanocapsules time-dependent. Notably, and unlike brain endothelial cells, statistically significant higher uptake of magnetic nanocapsules in HBVP cells was evidenced in the presence of an external magnetic field after only 3 h (Fig. 4b-d). This statistically significant increase in nanocapsules internalization was 1.49-fold and 2.06-fold after 3 and 24 h exposure, respectively.

Distinct magnetic responsiveness of a single nanocarrier depending on the cell type as observed herein have already been reported [65, 66]. However, in these cases, the magnetic targeting was deemed to be more efficient in brain endothelial cells than in U251 glioma cells. This might well be related to the replication rate of each cell type following the trend that the higher the replication rate of a cell type, the least the sensitivity to magnetic targeting. The results shown in this study support this conclusion in the sense that higher exposure time to the external magnetic field was needed to observe significant increases in cellular internalization of magnetic nanocapsules for the hCMEC/D3 cell line, the one with the highest replication rate. The not statistically different behavior in terms of magnetic targeting efficiency between brain endothelial and U87 glioma cells observed by Cui et al. might be accounted for by the low strength of the applied magnetic field and the low saturation magnetization of the magnetic PLGA nanoparticles [9].

## Conclusion

Lipid-based magnetic nanocapsules with a 12 wt.% iron oxide content have been obtained by a low-energy method upon the inclusion of oleic acid-coated magnetite nanoparticles within the oily core of lipid nanocapsules. These magnetic nanocapsules showed superparamagnetic-like behavior, which makes them suitable for biomedical application since for superparamagnetic materials there is no remanent magnetization upon removal of the external magnetic field. Magnetic nanocapsules showed high biocompatibility against both human cerebral endothelial cells and pericytes. Notably, upon exposure to an external magnetic field these magnetic nanocapsules significantly increased their in vitro targeting ability in both cerebral endothelial cells and pericytes. The exposure time needed to achieve this enhanced targeting ability was lower for pericytes, demonstrating that the magnetic targeting efficiency can also depend on the internalization rate of each cell type within the NVU. Altogether, the results on the interaction of magnetic nanocapsules with the human brain microvascular endothelial cells and with the human brain vascular pericytes highlight the potential of this formulation for magnetic targeting to the BBB.

2D monoculture models present some limitations to assess BBB targeting, since these models lack some of the features of BBB. Hence, in future work, further insight should be gained by studying the uptake of magnetic nanocapsules upon exposure to an external magnetic field in more complex and physiologically relevant in vitro BBB models.

Moreover, future work should focus on encapsulating drug substances to test the efficacy of this physical targeting approach in a treatment context. Lipophilic drugs for the treatment of brain diseases are the more likely to benefit from encapsulation into magnetic nanocapsules. In this regard, previous work in encapsulation into lipid nanocapsules of drugs such as retinoic acid, calcitriol or prostaglandins for remyelination in the context of multiple sclerosis [51, 67, 68], or lipophilic chemotherapeutics or cannabinoids for the treatment of glioma [49, 69] may lead the field in the years to come.

## Supplementary Information

Below is the link to the electronic supplementary material.

Supplementary file1 Figure S1: Selected area electron diffraction (SAED) pattern according to raw data (blue). The obtained intensity profile is compared with a powder X-ray diffraction pattern calculated for the reference ICSD structure hematite (ICSD 15840, orange). (JPG 204 KB)

Supplementary file2 Figure S2: Blank nanocapsules characterization: a) Representative intensity distribution profile (%) of blank nanocapsules as a function of the hydrodynamic diameter (nm); b) Representative ζ-Potential (mV) distribution of blank nanocapsules. (JPG 165 KB)

Supplementary file3 Figure S3: a) Cell viability after 72 h of treatment with magnetic nanocapsules (10-500 µg/mL) on the human cerebral microvascular endothelial cell line hCMEC/D3, b) Cell viability after 72 h of treatment with magnetic nanocapsules (10-500 µg/mL) on the human brain vascular pericytes HBVP cell line. All results were normalized to untreated control. Statistical analysis was performed using one-way ANOVA and Dunnett multiple comparison test. ****: p < 0.0001. (JPG 186 KB)

## Data Availability

The data that support the findings of this study are available from the corresponding authors upon reasonable request.
