# Supplementary figures and images for: Development and characterization of lipid nanocapsules loaded with iron oxide nanoparticles for magnetic targeting to the blood–brain barrier
Source: Drug Deliv Transl Res. 2024 May 13;14(12):3494–511. doi: 10.1007/s13346-024-01587-w (PMC11499457; doi:10.1007/s13346-024-01587-w)

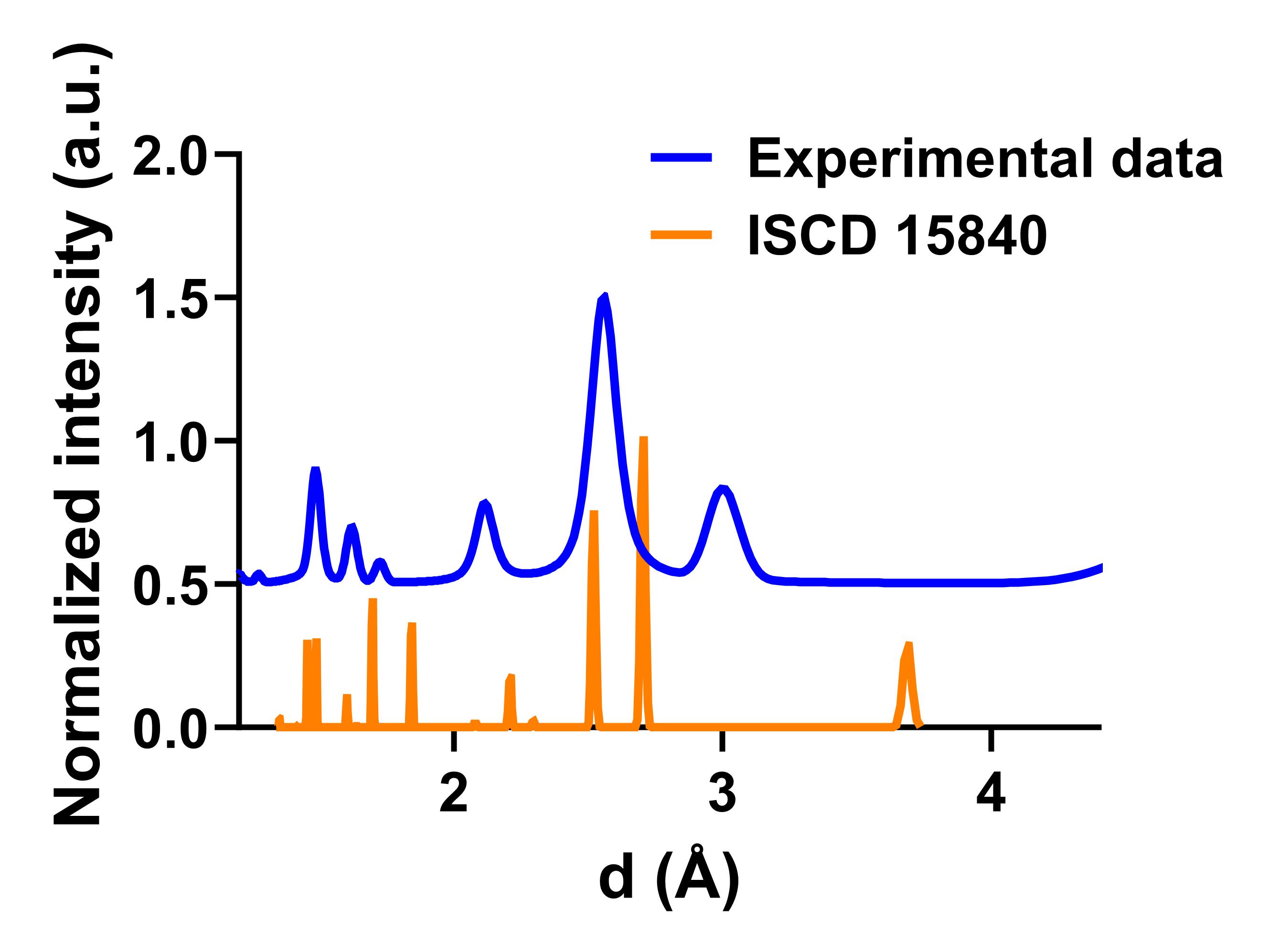

Supplement: Supplementary file 1 — Supplementary file1 Figure S1: Selected area electron diffraction (SAED) pattern according to raw data (blue). The obtained intensity profile is compared with a powder X-ray diffraction pattern calculated for the reference ICSD structure hematite (ICSD 15840, orange). (JPG 204 KB) [file 13346_2024_1587_MOESM1_ESM.jpg]

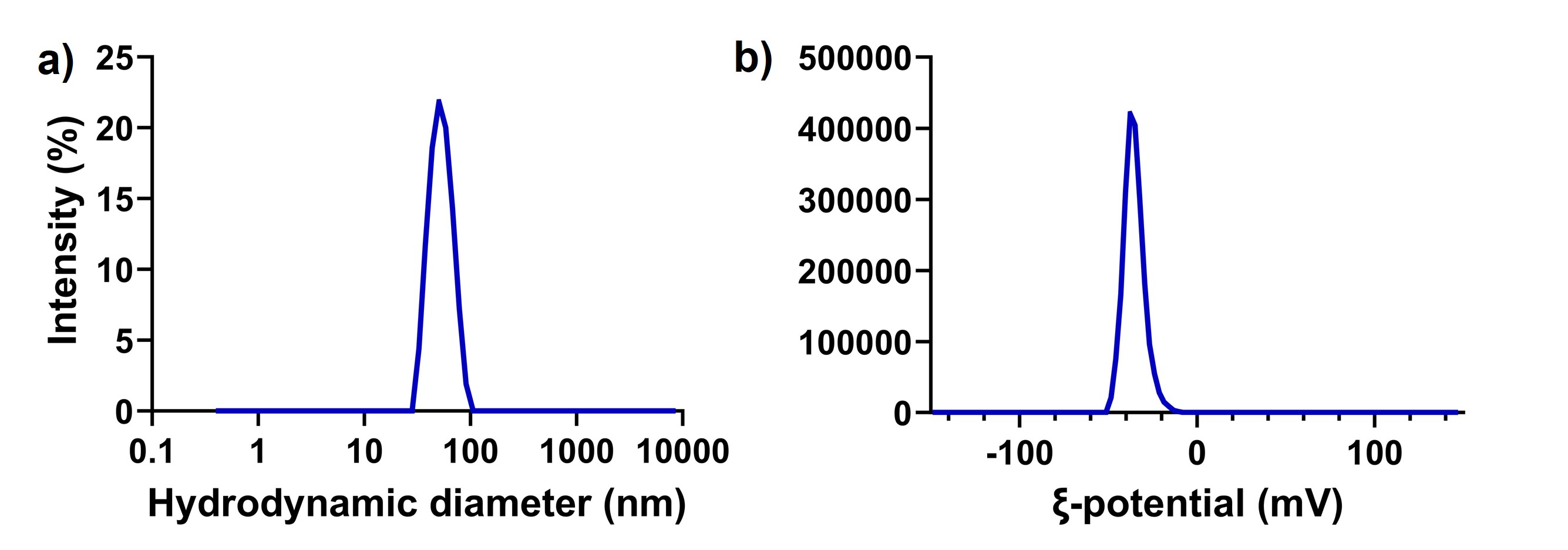

Supplement: Supplementary file 2 — Supplementary file2 Figure S2: Blank nanocapsules characterization: a) Representative intensity distribution profile (%) of blank nanocapsules as a function of the hydrodynamic diameter (nm); b) Representative ζ-Potential (mV) distribution of blank nanocapsules. (JPG 165 KB) [file 13346_2024_1587_MOESM2_ESM.jpg]

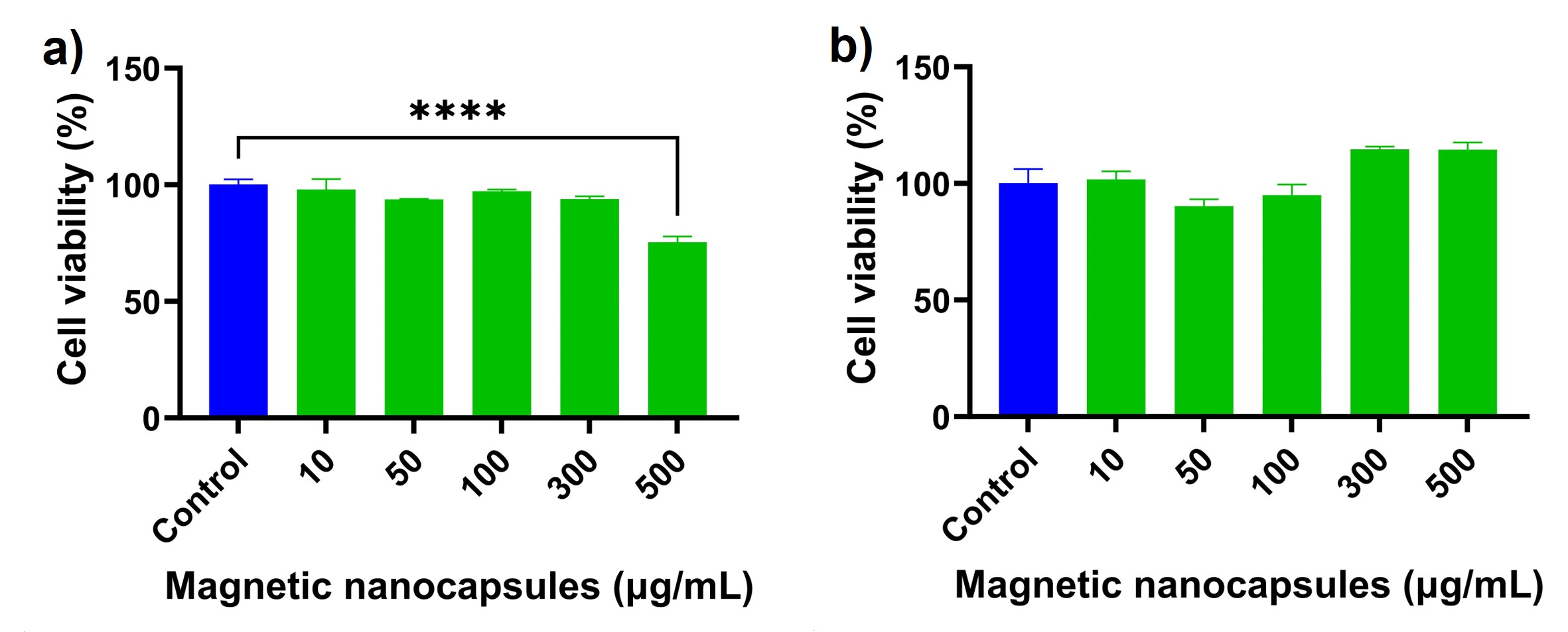

Supplement: Supplementary file 3 — Supplementary file3 Figure S3: a) Cell viability after 72 h of treatment with magnetic nanocapsules (10-500 µg/mL) on the human cerebral microvascular endothelial cell line hCMEC/D3, b) Cell viability after 72 h of treatment with magnetic nanocapsules (10-500 µg/mL) on the human brain vascular pericytes HBVP cell line. All results were normalized to untreated control. Statistical analysis was performed using one-way ANOVA and Dunnett multiple comparison test. ****: p < 0.0001. (JPG 186 KB) [file 13346_2024_1587_MOESM3_ESM.jpg]
